# From the West to the East: an evidence-based educational reform for modern medical students in traditional Chinese medicine learning

**DOI:** 10.3389/fmed.2023.1223614

**Published:** 2023-09-12

**Authors:** Zhenrui Liu, Zhixuan Ren, Linhan Fang, Yuxuan Liao, Dan Ren, Yao Yu, Yixuan Qin, Jianzhen Wu, Pengfei Rong

**Affiliations:** ^1^The Third Xiangya Hospital, Central South University, Changsha, China; ^2^Graduate School of Peking Union Medical College, Chinese Academy of Medical Sciences and Peking Union Medical College, Beijing, China; ^3^National Cancer Center, National Clinical Center of Cancer, Cancer Hospital, Chinese Academy of Medical Sciences and Peking Union Medical College, Beijing, China; ^4^Department of Traditional Chinese Medicine, Third Xiangya Hospital, Central South University, Changsha, China; ^5^Department of Radiology, The Third Xiangya Hospital, Central South University, Changsha, China

**Keywords:** modern medicine students, traditional Chinese medicine (TCM), education reform, teaching, medical education

## Abstract

**Introduction:**

Generally, Traditional Chinese Medicine (TCM) courses are now given to modern medicine students without proper course scheduling, resulting in poor teaching results.

**Methods:**

To analyze the main factors affecting TCM learning, we surveyed the medical students and TCM teachers from Xiangya School of Medicine of Central South University via online questionnaires. The questionnaire comprised two parts, the students' part included the basic information, the subjective cognition in TCM, the attitude toward TCM course arrangements, and the attitude toward curriculum content and the design of TCM. The teachers' part included the basic information, the attitudes and opinions on TCM course arrangements, and suggestions and views on TCM teaching reform. The related data were collected from 187 medical students divided into two groups, namely, clinical medical students and non-clinical medical students.

**Results:**

We found a more positive attitude toward TCM [including “Scientific nature of TCM” (*P* = 0.03) and “Necessity for modern medicine students to learn TCM” (*P* = 0.037)] in clinical medical students compared with non-clinical medical students, clinical and non-clinical medical students tended to find TCM courses difficult, and the students prefer clinical training to be better than theoretical teaching, while the teachers believe that lecture-based education should have a more significant proportion.

**Discussion:**

Hence, to optimize the current TCM teaching, we conducted education reform, including differentiated teaching, hybrid teaching, and selective teaching.

## 1. Introduction

Traditional Chinese medicine (TCM) has been practiced for around two thousand years and is composed mainly of acupuncture and herbal medicine (1). It is considered one of the oldest forms of healing (2), developing a holistic approach to diagnosis (e.g., using the Four Examinations), pathophysiology (using Yin-Yang and Five Phases), and physical therapy (such as massage). Despite its long history, many of the approaches used in TCM have been preserved and continue to be applied in modern medicine in China (3). It is also popular in Chinese culture circles worldwide (4). Internationally, it is estimated that between 60 to 75% of people with Chinese heritage in Taiwan, Japan, South Korea, and Singapore visit TCM practitioners at least once a year (5). Overall, TCM is an integral part of Chinese culture, representing a unique approach to health and healing. Modern medical students in China are required to study TCM and are encouraged to further develop its use by incorporating it with modern techniques (6).

TCM theories are significantly different from modern medicine as TCM is inextricably linked to ancient Chinese philosophies with strong cultural connotations. As a result of this undeniable disparity, it is difficult for a person educated in various cultures and medical systems to truly comprehend the nature of TCM, including indescribable characteristics of the Qi and Meridian Systems (7). Naturally, the discrepant perspectives and knowledge qualifications make it difficult for modern medical students to learn TCM effectively (8). However, learning Chinese medicine is still important to them. Firstly, TCM can effectively address the limitations of modern medicine, such as chronic pain and endocrine disorders. In addition, TCM focuses on the overall health of the patient and preventive care, rather than symptomatic and aftercare treatment in modern medicine, and understanding this helps modern medical students develop a more holistic professionalism. Modern medicine follows science, breaking down the human body into ever-smaller parts to study the profound mechanisms. As a result, all theories on anatomy, pathology, histology, physiology, genetics, and biochemistry have been gathered. However, TCM is based on practice and culture and has prominent features of dynamic, complex, fractal, and balanced/harmonious at system-level views (6). Due to the differences in thinking, it's hard for modern medical students to truly understand the fundamental theories of TCM (9). Issues such as lack of relevance for modern medical students, TCM theories needing to be more abstract, class hours needing to be longer for adequate understanding, and the separation of education theories from education practice still need to be solved.

Although the global promotion of TCM has the effect of providing alternative therapies for modern medicine, encouraging people to develop healthy lifestyles, etc. TCM education is now given to all medical majors without distinction in China, resulting in dissatisfaction among modern medical students (10). In China, modern medicine education is characterized and divided into clinical medicine, nursing, pharmacy (study the basic theory and basic knowledge of the main branches of pharmacy), clinical pharmacy (learn the basic knowledge and practical skills of pharmacy and clinical medicine), preventive medicine (mainly focuses on the essential knowledge and skills of etiology, prevention, screening, control, and eradication of infectious diseases and epidemics), preclinical medicine (especially study the basic theoretical knowledge of modern natural sciences and life sciences, basic medical disciplines, and generally master the basic knowledge of clinical medicine) (11). Different modern medicine majors have different professional requirements and occupational planning. Thus the differentiated teaching strategy should be considered instead of the present analogous scheme for better teaching results in TCM.

The TCM curriculum for modern medical students at The Third Xiangya Hospital of Central South University includes Yin-Yang, Five Phases, The Theory of Visceral Manifestations, Eight Principles, Four Examinations, Prescriptions, Traditional Chinese Herbs, Prevention and Treatment Principles, Acupuncture, and other topics. Several scholars of the teaching team have dedicated themselves to improving TCM education. However, issues such as lack of relevance for modern medical students, too abstract TCM theories, too short class hours for understanding and the separation of education theories from education practice still need to be solved. To improve our TCM education, we used questionnaires to assess the current learning situation of modern medical undergraduate students (5-year clinical medical students, 8-year clinical medical students, clinical pharmacy students, nursing students, preventive medicine students, and preclinical medicine students) at The Third Xiangya Hospital of Central South University. The questions covered subjective perception, level of satisfaction, and acquisitions.

To improve the learning effectiveness of TCM courses for modern medical students, we researched the advantages and disadvantages of the current teaching schedule. Based on the results, we designed several education reforms to optimize the teaching of TCM.

## 2. Methods

### 2.1. Ethical approval

The Ethics Association of The Third Xiangya Hospital of Central South University approved this study and all participants signed the informed consent form according to the requirements of the Ethics Association. The patients/participants provided their written informed consent to participate in this study.

### 2.2. Study design

We conducted a cross-sectional study to examine the attitudes of students majoring in modern medicine toward the TCM curriculum, using the method of convenience sampling survey. In addition, we collected the attitudes and opinions of teachers from the TCM teaching and research section of The Third Hospital Xiangya of Central South University for further analysis. The student-side questionnaire consisted of four sections: (1) demographic information of the participants, (2) degree of recognition toward the TCM curriculum, (3) evaluation of the current teaching mode for TCM, and (4) suggestions for improving the TCM curriculum. The initial part of the questionnaire comprised a demographic survey that gathered information on participants' academic backgrounds, including their grades and specific majors, as well as their learning objectives for TCM and their subjective perceptions of the TCM curriculum's difficulty. The second part assessed the degree of recognition medicine students had for the TCM curriculum. It used a five-point Likert scale to measure their scientific views on TCM knowledge and the extent to which they accepted the TCM curriculum. The third and fourth part aimed to identify problems with the current teaching model, examining teaching methods, the number of class hours, and the timing of the course. The teacher-side questionnaire focused on gathering basic information about the teachers, their attitudes toward teaching reform, and their suggestions for educational reform. Based on the findings of this survey, a teaching reform plan was developed.

### 2.3. Questionnaire distribution and recovery

We designed two questionnaires for students and teachers, respectively. Among the participants in the student-side questionnaire, the study comprises two distinct groups: clinical and non-clinical. The clinical group consists of individuals who have completed either the 5-year program of clinical medicine or the 8-year program of clinical medicine. The non-clinical group comprises individuals who majored in preclinical medicine, clinical pharmacy, preventive medicine, and nursing. The teacher-side questionnaires were distributed to teachers from the TCM teaching and research section of The Third Xiangya Hospital of Central South University. In October 2022, the student- and teacher-side questionnaires were distributed to all participants online via the Wenjuanxing platform (https://www.wjx.cn/). We collected seven teacher-side questionnaires and 187 student-side questionnaires, 187 of which (87 from clinical medical students and 100 from non-clinical medical students) with reliable and valid answers were used for research.

### 2.4. Questionnaire reliability analysis

To ensure the relevance and professionalism of the initial questionnaire to the actual situation, the two questionnaires were reviewed by two TCM educators, a statistical expert, and an official in charge of general teaching work in The Third Xiangya Hospital of Central South University. We also pretested the survey with twenty students and one teacher and modified the questionnaire based on the feedback. The Cronbach's coefficient (0.857) was used in the student-side questionnaire to test the internal consistency reliability.

### 2.5. Analysis of questionnaire data

SPSS.26.0 was applied for data analysis. The Kolmogorov–Smirnov test analyzed the distribution of 187 data collected from the students' side, a non-parametric test was used for the data following a skewed distribution, including the scientific nature of TCM, the necessity for modern medical students to learn TCM, the rationality of the course arrangement, degree of difficulty in learning TCM, and rationality of the class hours. The chi-square test was used to compare answers toward “Do you intend to take TCM elective course in the future?" and “Has TCM promoted other specialty courses or not?" between clinical and non-clinical medicine students. Therefore, independent sample *T*-test, chi-square analysis, and other methods were used to analyze the data to compare the differences between the two groups of data, including the goals of the clinical medicine students and non-clinical medicine students of subjects for the learning of TCM courses and the degree of recognition of the courses of TCM and attitudes toward the current teaching arrangement of TCM, with a *P*-value < 0.05 indicating a statistically significant difference. The teacher-side questionnaire analyzes teachers' attitudes and biases toward teaching arrangements and outcomes. The results were used as a reference for subsequent teaching reform plans.

## 3. Results

### 3.1. Questionnaire distribution and basic information

After half a month, 187 questionnaires were collected from 87 clinical medical students, including 64 questionnaires (34.22%) from 5-year clinical medical students and 23 questionnaires (12.3%) from 8-year clinical medical students. And there were 100 non-clinical medical students, including 25 clinical pharmacy students (13.37%), 23 nursing students (12.3%), 40 preventive medicine students (21.39%), and 12 clinical preparatory students (6.42%). The grades (from 3rd to 8th), the majors, and genders were presented in Table 1 as basic information.

**Table 1:** The basic information of students' side.

**Majors**		**Grades**			**Gender**	
		**3rd**	**4th**	**5th–8th**	**Male**	**Female**
5-years clinical medicine		52 (81.3%)	10 (15.6%)	2 (3.1%)	30 (46.9%)	34 (53.1%)
8-years clinical medicine		1 (4.3%)	2 (8.7%)	20 (87.0%)	13 (56.5%)	10 (43.5%)
Clinical pharmacy		14 (56.0%)	11 (44.0%)	0	10 (40.0%)	15 (60.0%)
Nursing		16 (69.6%)	7 (30.4%)	–	4 (17.4%)	19 (82.6%)
Preventive medicine		29 (72.5%)	5 (12.5%)	6 (15.0%)	16 (40.0%)	24 (60.0%)
Preclinical medicine		2 (16.7%)	7 (58.3%)	3 (25.0%)	4 (30.8%)	9 (69.2%)

To advance our research, we have procured seven completed teacher questionnaires from the TCM teaching and research section of The Third Xiangya Hospital of Central South University. The basic personal information of the instructor is outlined in Table 2. Furthermore, data analysis indicates that the instructor generally exhibits a favorable disposition toward incorporating the TCM curriculum for modern medical students.

**Table 2:** The basic information of teachers' side.

**Basic characteristics**	**Number (%)**
Title	
Lecturer	2 (28.57%)
Associate professor	5 (71.43%)
Length of employment	
< 5	1 (14.29%)
11–15	2 (28.57%)
16–20	1 (14.29%)
>20	3 (42.86%)
Education background	
Bachelor	1 (14.29%)
Master	2 (28.57%)
Doctor	4 (57.14%)
Attitude toward modern medicine students study TCM	
Very unsupportive	0 (0%)
Unsupportive	0 (0%)
Uncertain	2 (28.57%)
Supportive	1 (14.29%)
Very supportive	4 (57.14%)

### 3.2. The subjective attitudes and opinions on course arrangements of the students toward TCM

**Figure 1:**
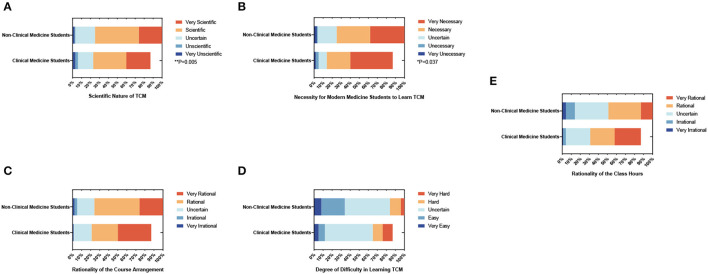
The percentage stacking histograms of the students' subjective attitudes and opinions on course arrangements toward TCM with a 5-point Likert scale. **(A)** Scientific nature of TCM. **(B)** Necessity for modern medical students to learn TCM. **(C)** The rationality of the course arrangement. **(D)** Degree of difficulty in learning TCM. **(E)** The rationality of the class hours. The nonparametric test was applied for the comparison.

A Likert scale was used to score five questions to investigate the subjective attitudes and opinions on course arrangements of clinical and non-clinical medicine students toward TCM (Table 3). Results showed that non-clinical students believed TCM to be more scientific (*P* = 0.005, Figure 1A) and deemed it more necessary to learn (*P* = 0.037, Figure 1B) than clinical medicine students. Most students regarded the course arrangement as rational (66% of clinical medical students and 76% of non-clinical students, Figure 1C) and the class hours as appropriate (64% of clinical students and 70% of non-clinical students, Figure 1E). Surprisingly, more than half of students found TCM hard to learn (59.7% of clinical medical students and 56%, Figure 1D). TCM is closely related to Chinese culture, so modern medical students may need to help grasp it.

**Table 3:** The subjective attitudes and opinions on course arrangements of the students toward TCM.

**Questions**	**Clinical medicine**	**Non-clinical medicine**	***P*-value**
	**Students (** *n* **1 = 87)(%)**	**Students (** *n* **2 = 100)(%)**	
Scientific nature of TCM			
Very unscientific	3 (3.4%)	2 (2.0%)	**0.003** ^**^
Unscientific	3 (3.4%)	1 (1.0%)	
Uncertain	17 (19.5%)	22 (22.0%)	
Scientific	37 (42.5%)	49 (49.0%)	
Very scientific	27 (31.0%)	26 (26.0%)	
Necessity for modern medicine students to learn TCM			
Very unnecessary	2 (2.3%)	3 (3.0%)	**0.037** ^*^
Unnecessary	3 (3.4%)	1 (1.0%)	
Uncertain	9 (10.3%)	21 (21.0%)	
Necessary	26 (29.9%)	37 (37.0%)	
Very necessary	47 (54.0%)	38 (38.0%)	
Very scientific	27 (31.0%)	26 (26.0%)	
Rationality of the course arrangement			
Very irrational	1 (1.1%)	2 (2.0%)	0.104
Irrational	0 (0.0%)	3 (3.0%)	
Uncertain	20 (23.0%)	19 (19.0%)	
Rational	29 (33.3%)	50 (50.0%)	
Very rational	37 (42.5%)	26 (26.0%)	
Degree of difficulty in learning TCM			
Very easy	5 (5.7%)	8 (8.0%)	0.170
Easy	7 (8.0%)	26 (26.0%)	
Uncertain	23 (26.4%)	10 (10.0%)	
Hard	41 (47.1%)	32 (32.0%)	
Very hard	11 (12.6%)	24 (24.0%)	
The rationality of the class hours			
Very irrational	1 (1.1%)	4 (4.0%)	0.219
Irrational	3 (3.4%)	10 (10.0%)	
Uncertain	27 (31.0%)	37 (37.0%)	
Rational	27 (31.0%)	36 (26.0%)	
Very rational	29 (33.3%)	13 (13.0%)	

The nonparametric test was applied for the comparison. *P* < 0.05 is indicated in bold. ^*^P < 0.05. ^**^P < 0.01. Clinical-medicine students (*n*1 = 87) and Non-clinical medicine students (*n*2 = 100) are included. TCM, traditional Chinese medicine.

### 3.3. The subjective attitudes of the students toward TCM curriculum content and design

Furthermore, we surveyed the subjective attitudes toward TCM curriculum content and design in clinical and non-clinical medical students (Table 4). The results showed that more non-clinical students thought basic theories of TCM were challenging (34.5% of clinical medical students and 54.0% of non-clinical medical students), which might be associated with the lack of clinical practice.

**Table 4:** The subjective attitudes toward TCM curriculum content and design in clinical medicine and non-clinical medicine students.

**Questions**	**Clinical medicine**	**Non-clinical medicine**
	**Students (** *n* **1 = 87) (%)**	**Students (** *n* **2 = 100) (%)**
The hardest part in TCM learning		
Basic theories of TCM	30 (34.5%)	54 (54.0%)
Clinical basis of TCM	25 (28.7%)	29 (29.0%)
Others	32 (36.8%)	17 (17.0%)
The most appropriate grade to learn TCM		
1st grade	8 (9.2%)	9 (9.0%)
2nd grade	14 (16.1%)	19 (19.0%)
3rd grade	37 (42.5%)	39 (39.0%)
4th grade	14 (16.1%)	7 (7.0%)
Indifferent	13 (14.9%)	17 (17.0%)
Others	1 (1.1%)	1 (1.0%)
Favorite teaching methods of TCM (multiple choice)		
Problem-based learning (PBL)	29 (11.6%)	16 (6.9%)
Case-based learning (CBL)	40 (16.1%)	23(10.0%)
Team-based learning (TBL)	22 (8.8%)	18 (7.8%)
Role play	25 (10.0%)	20 (8.7%)
Clinical training	65 (26.1%)	69 (29.9%)
Lecture-based teaching	33 (13.3%)	49 (21.2%)
Online learning	15 (6.0%)	28 (12.1%)
Knowledge contest	16 (6.0%)	4 (1.7%)
Others	4 (1.6%)	4 (1.7%)
Achieved goals during the study of TCM (multiple choice)		
Understood the basic theories of TCM	75 (20.9%)	85 (22.4%)
Heighten the interest of TCM	71 (19.8%)	71 (18.7%)
Use TCM in daily life	43(12.0%)	54 (14.2%)
Facilitated the learning of TCM	41 (12.0%)	49 (14.2%)
Deepen the love of traditional Chinese culture	58 (16.2%)	66 (17.4%)
Inspired scientific research ideas	23 (6.4%)	16 (4.2%)
Passed the examinations	45 (12.5%)	37 (9.7%)
Others	3 (0.8%)	2 (0.5%)

Basic theories of TCM includes Yin And Yang Five Elements Doctrine/The Theory of Visceral Manifestations/Five- Viscera, etc.

Clinical basis of TCM includes Principles of Prevention and treatment/Eight Principals/Four Diagnoses/Etiology, etc. Others includes Tradition Chinese Medicine/Prescriptions/Acupuncture, etc.

According to the schedule, modern medical students should take TCM in the 3rd grade. To figure out the most appropriate age to learn TCM, we collected data from all the modern medical students of The Third Xiangya Hospital. Gratifyingly, it was found that most students (42.5% of clinical medical students and 39.0% of non-clinical medicine students) considered the 3rd grade the most suitable time to learn TCM.

The study also researched students' preferences for various teaching methods. Both clinical medicine students and non-clinical medicine students preferred clinical training (26.1% of clinical medical students and 29.9% of non-clinical medical students). However, clinical medical students selected case-based learning (CBL; 16.1%), while non-clinical medical students preferred lecture-based teaching (21.2%).

Several presumed able options were set to compare the learning outcomes between clinical and non-clinical medical students. Results showed that more clinical medicine students chose “Inspired scientific research ideas" (6.4% of clinical medical students and 4.2% of non-clinical medical students) and “Passed the exam" (12.5% of clinical medical students and 9.7% of non-clinical medical students) than non-clinical medical students. In contrast, more non-clinical medical students chose “Understood the basic theories of TCM" (20.9% of clinical medical students and 22.4% of non-clinical medical students) and “Used TCM in daily life" (12.0% of clinical medical students and 14.2% of non-clinical medical students) than clinical medical students. The outcome demonstrated that clinical medical students were likely to integrate theories with practice, and non-clinical students had the disposition to concentrate on basic theories.

### 3.4. The subjective attitudes and opinions on course arrangements of the teachers toward the TCM curriculum

To improve teaching effectiveness, we designed questions to gather teachers' attitudes toward current teaching methods and models in the TCM curriculum (Table 5). The role play was the most effective teaching method, as most respondents (71.4%) indicated. Regarding the teacher's preference for practical or theoretical teaching, the survey revealed that 57.1% preferred lecture-based teaching, while 42.9% perceived no significant difference. Thus, educators generally preferred theoretical teaching methods. Moreover, the survey results revealed that teachers were generally satisfied with the current teaching materials and teaching evaluation mechanisms; responses ranged from “Uncertain" (28.6%) to “Satisfied” (71.4%). However, teachers remained neutral in their attitudes toward allocating course hours. These findings suggested that teachers held varying attitudes toward specific teaching methods and models while generally expressing satisfaction with the overall teaching arrangements. Nevertheless, the neutral stance toward course hour allocation warranted further investigation.

**Table 5:** The subjective attitudes and opinions on course arrangements of the teachers toward the TCM curriculum.

**Questions**	**Numbers (%)**
The best way to teach TCM (multiple choice)	
Problem-based learning (PBL)	4 (57.14%)
Case-based learning (CBL)	3 (42.86%)
Role play	5 (71.43%)
Clinical training	3 (42.86%)
Lecture-based teaching	4 (57.14%)
Inclination to teach basic knowledge in textbooks or teaching in practice	
Teaching basic knowledge in textbooks	4 (57.14%)
Teaching in practice, both are important	0 (%)
There is no absolute difference.	3 (42.86%)
Satisfaction with the teaching materials currently using	
Very dissatisfied	0 (0%)
Dissatisfied	0 (%)
Uncertain	2 (28.57%)
Satisfied	5 (71.43%)
Very satisfied	0 (%)
Satisfaction with the current teaching evaluation mechanisms	
Very dissatisfied	0 (%)
Dissatisfied	0 (%)
Uncertain	2 (28.57%)
Satisfied	5 (71.43%)
Very satisfied	0 (%)
Satisfaction with TCM class hours	
Very dissatisfied	0 (%)
Dissatisfied	2 (28.57%)
Uncertain	3 (42.86%)
Satisfied	2 (28.57%)
Very satisfied	0 (%)

### 3.5. The suggestions and opinions of teachers on TCM teaching reform

To facilitate subsequent teaching reforms, several questions were designed to investigate the teachers' attitudes and opinions on teaching reform in TCM (Table 6). It revealed that the shortage of class hours and dense course arrangements (85.7%) and the difficulty in training TCM thinking (100%) were the most pressing issues in the current TCM teaching process. Flipped classroom (57.1%), CBL teaching (57.1%), trainee practice training (57.1%) and heuristic teaching (71.4%) were the teaching methods most supported by teachers. Additionally, the teachers also generally supported cross-disciplinary learning between TCM and other subjects. Diagnostics was the subject most closely related to TCM learning for 85.7% of teachers, followed by internal medicine at 71.4%. In terms of interactive teaching, teachers believed it was necessary to increase the interactive process in the classroom and support receiving feedback from students after teaching. Finally, the teachers kept teaching TCM in the third year (57.1%) and considered expanding class hours and adjusting teaching objectives necessary. Based on the current challenges in TCM teaching, teachers generally supported the proposed teaching reforms while maintaining a reserved attitude toward some aspects of maintaining the current state.

**Table 6:** The teachers' suggestions and views on TCM teaching reform.

**Questions**	**Number (%)**
The problems of TCM teaching (multiple choice)	
Less class time and intensive course schedule	6 (85.71%)
Students are not very interested	1 (14.29%)
Difficult to understand the content and the cultivation of TCM thinking	7 (100%)
Paid more attention to the exam than other formative evaluations	1 (14.29%)
Lack of a comprehensive feedback after assessment	1 (14.29%)
Medical knowledge differs from major to major	2 (28.57%)
Lack of the ability to understand the classical Chinese involved in the course	3 (42.86%)
Favorite teaching models and methods (multiple choice)	
PAD class (exchanging the roles of teachers and students)	2 (28.47%)
Flipped classroom (shifting the decision to learn from the teacher to the student)	4 (57.14%)
Online and offline hybrid teaching	1 (14.29%)
Problem-based learning (PBL)	3 (42.86%)
Case-based learning (CBL)	4 (57.14%)
Task-based learning (TBL)	2 (28.57%)
Role play	4 (57.14%)
Clinical training	5 (71.43%)
Heuristic education	
Which courses do you think the study of TCM helpful for learning (multiple choice)	
Pharmacology	3 (42.86%)
Diagnostics	6 (85.71%)
Pathology	1 (14.29%)
Internal medicine	5 (71.43%)
Surgery	1 (14.29%)
Basic nursing	2 (28.57%)
Internal medicine	2 (28.58%)
Rehabilitation nursing	2 (28.59%)
Natural medicinal chemistry	1 (14.29%)
Pharmacy	1 (14.30%)
Whether to support the cross-learning of TCM and other medical courses	
Very unsupportive	0 (0%)
Unsupportive	0 (0%)
Uncertain	2 (28.57%)
Supportive	2 (28.58%)
Very supportive	3 (42.86%)
When is the most suitable grade for the TCM course	
1st grade	0 (0%)
2nd grade	1 (14.29%)
3th grade	4 (57.14%)
4th grade	2 (28.47%)
What measures can be taken to optimize the class hour arrangement (multiple choice)	
Increase teaching hours	4 (57.14%)
Adjust teaching objectives and refine course content	6 (85.71%)
Adjust the proportion of each teaching mode in the process	3 (42.86%)
Offer elective courses	2 (28.57%)
Necessity of receiving teaching feedback	
Very unnecessary	0 (0%)
Unnecessary	0 (0%)
Uncertain	0 (0%)
Necessary	4 (57.14%)
Very necessary	3 (42.86%)
The necessity of adding more interactive process during lecture-based teaching	
Very unnecessary	0 (0%)
Unnecessary	1 (14.29%)
Uncertain	0 (0%)
Necessary	2 (28.57%)
Very necessary	4 (57.14%)

## 4. Discussion and education reform

### 4.1. Differentiated teaching reform strategy: how to formulate differential TCM curriculum for clinical and non-clinical medical students

Based on the survey results, clinical medical students were more likely to learn TCM and acquired more knowledge from TCM than non-clinical medical students. We attributed this result to three possible factors. Clinical medical students tend to use their TCM knowledge in clinical work. In contrast, non-clinical medical students barely use TCM knowledge at work, clinical medical students tend to focus on clinical treatment and prioritize clinical efficacy, but non-clinical medicine students tend to establish logical thinking based on fundamental medical research, and the differences in clinical practice experience and course arrangements between clinical and non-clinical medicine students explained the reason for the differences subjective attitudes toward TCM (Supplementary Table 1) (12). By integrating theoretical knowledge with clinical practice, clinical medical students can deepen their understanding of TCM through clinical training and other courses. Previous studies have used the abilities required by healthcare professionals as a guide to design curricula for most healthcare education programs (13). Therefore, we recommend customizing teaching plans for clinical and non-clinical medical students, dividing them into two different streams. For clinical medical students, increasing their clinical practice training, especially in evidence-based practice (14), can help them better understand the clinical applications of TCM and enhance their confidence in clinical practice (15). For non-clinical medical students, emphasis should be placed on TCM's basic theory and cultural background. However, it is essential to note that our non-clinical group includes disciplines such as anesthesiology and nursing, which will be exposed to clinical practice in the future. Therefore, more detailed teaching arrangements are necessary according to specific professional requirements.

### 4.2. Teaching model reform strategy: how to improve the enthusiasm and effectiveness of clinical and non-clinical medical students in TCM learning

According to the results, clinical and non-clinical medical students tended to find TCM courses difficult. This may be due to the fundamental differences between TCM and medicine, as they have different conceptual foundations (16). Medical students may need help understanding the TCM theory and diagnostic process, leading to a lack of enthusiasm and effectiveness in learning the course (17). To improve the excitement and energy of clinical and non-clinical medical students in TCM learning, we suggest implementing teaching reforms in two areas: expanding cultural background teaching and diversifying teaching methods. In terms of cultural background teaching, TCM has a history of thousands of years and plays a crucial role in treating various diseases (18). Therefore, understanding TCM's cultural background and development is essential for teaching this course. Students with a deeper cultural understanding of TCM will better understand and apply TCM concepts and theories (19). Thus, we suggest supplementing TCM teaching with cultural and ideological education. Regarding teaching methods, our survey indicated that medical students find the current TCM course arrangement reasonable but differ in their attitudes toward teaching methods. We recommend diversifying teaching methods to improve medical students' enthusiasm and effectiveness in learning TCM (20). Four possible areas for reform are as follows: (1) increasing the analysis of TCM case studies to guide evidence-based clinical practice (14); (2) promoting interactive teaching through questioning, discussion, and group activities; (3) enriching practical activities such as visits to TCM hospitals and traditional Chinese medicine plantations and conducting TCM diagnostic and treatment practical activities; (4) utilizing modern technology such as multimedia and virtual simulation.

### 4.3. Adaptive teaching reform strategy: how to determine the teaching method according to the different courses in TCM

The study identified differences in the preferred teaching mode and teaching methods between the students and the teachers. The students prefer clinical training to be better than theoretical teaching, while the teachers believe that lecture-based education should have a more significant proportion (Tables 4, 6). However, the study found that students considered the main learning objective to be mastering theoretical knowledge. Therefore, there is a need to adjust the teaching methods to better align with the student's learning objectives. This can be achieved by compromising and identifying the most suitable teaching method matching content. The results identified differences in the preferred teaching mode and teaching methods between the students and the teachers. To settle this divergence, we can choose teaching methods according to the contents. Since PBL was introduced in Chinese medical education for a long, it has been widely used in medical schools (21). PBL is one of the best-described interactive learning methods. It is advocated by many as more effective in terms of lifelong learning skills and as being more fun (22), so when teaching Yin-Yang and Five Phases, we can use PBL to make it enjoyable to learn and encourage students to explore. CBL encourages the community-based, student-centered, patient-oriented exploration of realistic and specific situations (23). Teachers can adopt CBL teaching when teaching TCM formulas to deepen the understanding of efficacy and range of use based on actual cases. Role-playing provides a positive learning experience (23) and brings students the achievement of decision-making skills (24) and the promotion of reflection and self-efficacy (25). When teaching Four Examinations, students can role-play under the teacher's guidance, which allows students to get the hang of using the theories. What's more, we designed an After Probation Review sheet for TCM teaching, which would help students review the content of the clinical training class, thus have a better grasp of the specific TMC clinical skills (Supplementary Data Sheet 1). In conclusion, the research identifies differences between the students and the teachers regarding their preferred teaching modes and methods. It also suggests that adjustments can be made to better align teaching goals and learning objectives. Furthermore, students support the continued teaching of TCM in later stages of study (Table 5). Still, more research is needed to determine the most effective teaching.

## 5. Conclusion

This was the first study to explore modern medical students' subjective cognition and satisfaction with the current TCM education. Meanwhile, we also investigated teachers' opinions. We also found discrepancies between clinical and non-clinical medical students, which was valuable and helpful in blazing new trails during the medical education reform. Clinical medical students tended to understand the diagnosis and treatment of TCM in common diseases. In contrast, non-clinical medicine students tended to have common knowledge of TCM in health care and prevention. On the part of teachers, a general analysis of the problems in various aspects of the current teaching of traditional Chinese medicine was carried out. A confident attitude was shown, follow-up teaching reform investigations were conducted based on these problems, and more scientific education reform-specific measures were analyzed. The main direction of follow-up is to adjust the corresponding number of class hours and add relatively new teaching methods to the teaching process to achieve better teaching effects. Then, differentiated teaching is carried out according to the different natures of students' majors so that they can accomplish the other teaching goals of corresponding majors and realize long-term teaching development.

**Figure 2:**
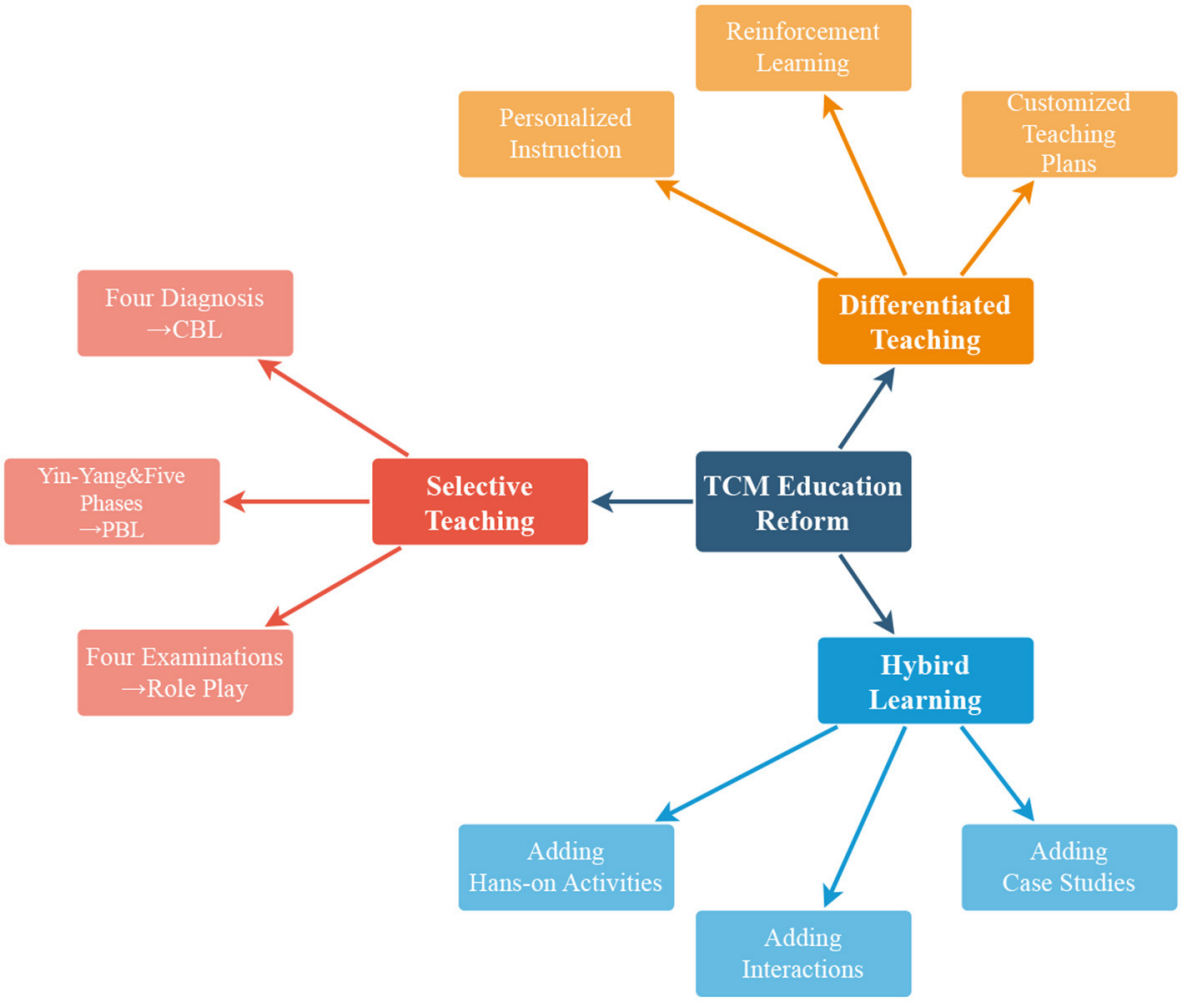
The total conclusion of TCM education reform.

We aim to conduct a design study on the teaching reform of TCM courses in medical universities and analyze appropriate teaching reform measures to promote TCM education. There are differences in professional requirements, basic thinking, and professional clinical practice environments between clinical and non-clinical medical students. According to the results, the TCM education reform comprised three main parts: differentiated teaching, hybrid teaching, and selective teaching (Figure 2). Differentiated teaching can meet the differences in professional requirements, hybrid teaching can make it easier for modern medical students to learn TCM, and selective teaching can optimize the teaching results.

Meanwhile, we found differences between teachers and students regarding certain aspects of teaching, which suggests that a comprehensive analysis of both attitudes is necessary to achieve the unity of teaching goals and learning objectives in the teaching reform. In summary, to improve the shortcomings of TCM education in medical universities, promote the coordinated development of TCM and medicine, and meet the basic teaching requirements of medical students, we should further improve the TCM teaching system and develop teaching models that match the needs of different medical majors.

## Data availability statement

The original contributions presented in the study are included in the article/Supplementary material, further inquiries can be directed to the corresponding authors.

## Author contributions

ZL, ZR, and DR: questionnaire design and questionnaire survey. ZL and ZR: data sorting, statistical analysis, and manuscript writing. YL: manuscript revision and paper conception. YY and YQ: manuscript revision.
