# Supplementary material for: From the West to the East: an evidence-based educational reform for modern medical students in traditional Chinese medicine learning
Source: Front Med (Lausanne). 2023 Sep 12;10:1223614. doi: 10.3389/fmed.2023.1223614 (PMC10520962; doi:10.3389/fmed.2023.1223614)
Supplement: Supplementary file 2 [file Data_Sheet_1.docx]

**AAR (After Action Review) sheet of TCM clinical training**

Name:

Date:

Team members:

**Step 1** Aims

**Step 2** Accomplishments of aims

**Step 3** Pros (pre-course preparations, clinical skills, time control, team collaboration, etc.)

**Step 4** Things that can be improved (pre-course preparations, clinical skills, time control, team collaboration, etc.)

**Step 5** Favorable or unfavorable events in training

**Events Circumstance**
